# Mineral-Mediated Oligoribonucleotide Condensation: Broadening the Scope of Prebiotic Possibilities on the Early Earth

**DOI:** 10.3390/life13091899

**Published:** 2023-09-12

**Authors:** Vincent S. Riggi, E. Bruce Watson, Andrew Steele, Karyn L. Rogers

**Affiliations:** 1 Rensselaer Astrobiology Research and Education Center, Rensselaer Polytechnic Institute, Troy, NY 12180, USA; watsoe@rpi.edu (E.B.W.); asteele@carnegiescience.edu (A.S.); rogerk5@rpi.edu (K.L.R.); 2 Department of Earth and Environmental Sciences, Rensselaer Polytechnic Institute, Troy, NY 12180, USA; 3 Earth and Planets Laboratory, Carnegie Institution for Science, 5251 Broad Branch Rd NW, Washington, DC 20015, USA

**Keywords:** prebiotic chemistry, polymerization, condensation, ribonucleic acid, mineralogy, adsorption, early Earth, Hadean

## Abstract

**Simple Summary:**

Although life on earth is quite diverse, some biological molecules are common across all life forms, both extant and extinct, and thus are thought to have been present as life emerged. Identifying how such compounds could have formed prior to life is therefore a critical step in understanding the origin of life and the potential for life elsewhere in the solar system and beyond. One class of biomolecule crucial to modern life are nucleic acids, which carry the genetic code and are integral to cellular replication and function. In modern biology, cellular machinery synthesizes these molecules; however, prior to life’s beginning, it is possible that naturally occurring minerals played a role in the synthesis and polymerization of these molecules. Only a few minerals have been tested thus far; here, we investigate a variety of minerals for their ability to promote elongation of ribonucleic acid in water. In doing so, both the minerals and their environments of formation are tested for their potential to promote elongation. We show that several newly tested minerals can promote synthesis, suggesting that a broader set of environments may have been able to host chemical reactions relevant to the origin of life than previously assumed.

**Abstract:**

The origin of life on earth requires the synthesis of protobiopolymers in realistic geologic environments along strictly abiotic pathways that rely on inorganic phases (such as minerals) instead of cellular machinery to promote condensation. One such class of polymer central to biochemistry is the polynucleotides, and oligomerization of activated ribonucleotides has been widely studied. Nonetheless, the range of laboratory conditions tested to date is limited and the impact of realistic early Earth conditions on condensation reactions remains unexplored. Here, we investigate the potential for a variety of minerals to enhance oligomerization using ribonucleotide monomers as one example to model condensation under plausible planetary conditions. The results show that several minerals differing in both structure and composition enhance oligomerization. Sulfide minerals yielded oligomers of comparable lengths to those formed in the presence of clays, with galena being the most effective, yielding oligonucleotides up to six bases long. Montmorillonite continues to excel beyond other clays. Chemical pretreatment of the clay was not required, though maximum oligomer lengths decreased from ~11 to 6 bases. These results demonstrate the diversity of mineral phases that can impact condensation reactions and highlight the need for greater consideration of environmental context when assessing prebiotic synthesis and the origin of life.

## 1. Introduction

Spontaneous condensation and polymerization of various biomolecules, including polynucleotides, polypeptides, and polysaccharides, are critical steps in the emergence of life from a sterile environment and are consequentially frequent topics of study [1,2,3]. In many cases, these syntheses are dehydration reactions and are more favorable under anhydrous conditions than in aqueous environments [4,5]. For example, standard molal Gibbs free energies of aqueous peptide bond formation tends to be positive at ambient temperature and pressure; synthesis only becomes favorable at elevated temperatures [4,6]. Similarly, aqueous polyribonucleotide synthesis from ribonucleotide monomers can be energetically unfavorable under some laboratory conditions [7,8,9]. In the case of ribonucleotide polymerization, the reaction may proceed if sufficient energy is made available, either through dissociation of an activating group or through cleavage of other high-energy bonds [10,11,12,13].

When the conditions that allow for condensation are met, yields are typically modest but can be enhanced significantly by the inclusion of inorganic catalysts or other reactants in the system [6,14,15]. In the cases of both peptides and oligonucleotides, clay minerals have been shown to promote condensation; this includes enhanced condensation of peptides in the presence of hectorite [16] and condensation of oligoribonucleotides in the presence of treated montmorillonite clay [15]. In both cases of experimental biopolymer synthesis, non-clay minerals are drastically underrepresented in experimental studies of condensation and polymerization. In this study, oligonucleotides are selected as a case study for prebiotic condensation reactions and the effects of various mineral groups on synthesis are explored. However, it should be noted that similar investigations can (and perhaps should) be applied to other polymeric molecular types, including polypeptides and polysaccharides.

In the case of oligonucleotides and RNA, montmorillonite is one of the most widely implemented and effective routes currently identified for enhancing polymerization abiotically [15,17,18,19,20]. However, in all reported cases, the montmorillonite is first chemically optimized via the “Banin process”—a multi-step chemical treatment in which the clay is suspended and stirred in a strongly acidic solution to remove interlayer cations before an anion exchange resin is used to replace the anion of the free acid with hydroxide, which reacts with free hydrogen in the acidic suspension to form water. An amount of salt (usually a sodium salt) determined by the clay’s exchange capacity is then added to produce a clay of the desired exchangeable cation composition [21,22]. In general, only those clays subjected to this or similar treatment have exhibited catalytic behavior. However, it is difficult to imagine a scenario on the early Earth in which this process would occur naturally given the precise pH conditions required for cation exchange and the inhibitory effects of excess aqueous salts expected in natural waters. Furthermore, not all montmorillonite samples are able to produce RNA oligomers (even after treatment via the Banin process) and the mechanism behind this observed behavior remains unclear [18].

Consideration should also be given to the geologic context of mineral formation to accurately assess the feasibility of any mineral as a potential contributor to prebiotic chemical synthesis. All minerals are inextricably associated with the environmental systems in which they form, and the mineralogical inventory of Earth has changed significantly through time [23]. Montmorillonite is a common product of various geological processes and is often found as a product of the aqueous alteration of basalt [24,25,26,27,28]. The nature of the Hadean crust and the primary rock available for weathering is somewhat uncertain due to a lack of a geologic record, except for extraordinarily ancient detrital zircons [29]. The nature of the Hadean crust was also likely dynamic, possibly evolving from a complex ultramafic composition to a basaltic composition by the late Hadean and accompanied by an early onset of plate tectonics [30]. Additionally, evidence from Hadean zircons demonstrates the presence of felsic igneous rock [31,32]. Accordingly, the mineral inventory available during the Hadean for alteration and potential prebiotic organic synthesis would comprise igneous rocks ranging from ultramafic through felsic compositions. The presence of montmorillonite prior to life is therefore possible, and perhaps even likely. Montmorillonite could also have been present early in the Hadean as a product of the aqueous weathering of chondritic material and would have increased in abundance as the crust evolved, though weathering products of ultramafic crust, such as saponite, have been suggested to dominate volumetrically relative to montmorillonite [33].

Although montmorillonite was likely available at that time, the focus of prebiotic experiments on montmorillonite is largely due to its experimental history preceding any implementation into prebiotic chemistry. Incorporation of montmorillonite was adopted as a consequence of early observations of the interactions between organic compounds and montmorillonite in modern terrestrial soils [34] and not due to its geological significance or abundance during the Hadean or early Archaean, when life is likely to have begun [35]. Prior to any suggestion of their potential relevance to astrobiology and prebiotic chemistry, clay minerals were studied for their ability to protect proteins and other large organic molecules from degradation in modern soils, as they are a notable source of nitrogen [36]. In the late 1930s and early 1940s, montmorillonite was singled out as one of the most effective clays for the adsorption and protection of proteins [37,38,39]. These observations motivated investigation into the potential effects of montmorillonite on prebiotic chemical reactions through adsorption and protection of prebiotic organic constituents [34]. Additionally, the “Clay Hypothesis” [40], which posits that life directly evolved from inorganic clay minerals, would later develop from an early idea suggesting that phyllosilicates are capable of a primitive mimic of biological evolution through the natural selection and propagation of crystal defects [41].

Suggestions such as these led to research that was focused almost exclusively on montmorillonite and inadvertently resulted in the vast majority of other clay and non-clay minerals being largely ignored. Rather than exploring the diverse plethora of minerals that were likely abundant in early Earth environments, montmorillonite was nearly exclusively implemented in the astrobiologically oriented experimental studies that followed. These included investigations of the adsorption of purines, pyrimidines, and nucleosides [42,43], catalysis of phosphate ester bond formation [44], and catalysis of dimerization of adenosine monophosphate (AMP) [45]. Eventually, polymerization of adenosine 5′-phosphorimidazolide in the presence of montmorillonite was demonstrated, which resulted in the first abiotically produced RNA oligomers catalyzed by a mineral [15].

Montmorillonite-focused studies continued beyond this point and included montmorillonite-driven Fischer–Tropsch-type synthesis of lipids [46] and catalysis of bilayer vesicle formation from fatty acid micelles [47]. Montmorillonite also became a “go-to” mineral for future abiotic RNA polymerization studies, including synthesis of longer RNA oligomers [48], polymerization following “in situ” nucleotide activation in aqueous solutions [19], and nucleotide selectivity during abiotic polymerization [20]. The narrow focus on montmorillonite is informative of the mechanism and specific parameters through which the mineral interacts with and contributes to abiotic chemical synthesis pathways but also limits the scope of knowledge gained regarding prebiotic synthesis to those environments in which montmorillonite is available, whereas the remainder of Earth’s vast mineralogical inventory remains largely untapped experimentally.

Although montmorillonite has nearly exclusively dominated experimental abiotic RNA polymerization studies, several additional minerals, including those within the zeolite family, have been proposed as likely candidates for the catalysis of RNA polymerization due to their high adsorptive capacity and organophilic properties; however, these remain untested [49,50]. Furthermore, a growing body of work on nucleotide adsorption onto various mineral surfaces suggests that RNA–mineral interactions, and possibly polymerization, occur under a broader set of conditions than merely those that include montmorillonite [51,52]. For example, Biondi et al. [53] demonstrated a strong tendency for RNA to adsorb onto carbonate minerals, among others. Furthermore, catalysis of RNA polymerization has recently been reported to occur in the presence of glasses of various compositions, demonstrating that promotion of condensation by a solid phase is not exclusively a feature of montmorillonite [54].

Demonstrating enhanced polymerization in the presence of a variety of mineral phases (in addition to montmorillonite) would bolster the possibility of various biopolymers’ involvement in the origin of life by expanding the potential environments on the early Earth that can host prebiotic chemical synthesis. An attempt to show the catalytic effect of various other minerals, including non-clay minerals, was made by Miyakawa et al. [55]. However, the only notable catalytic effect documented was the dimerization of activated nucleotides in the presence of galena. Whereas this study was an important step towards widening the range of minerals explored experimentally, the large majority of naturally occurring minerals remain untested for potential influence over prebiotic synthesis reactions. Therefore, we conducted a series of experiments to assess the potential for a variety of different minerals and mineral groups, including sulfides, clays, and other silicates to enhance abiotic RNA polymerization in aqueous solutions (a comprehensive list of minerals is shown in Table 1). The results from all experiments were then used to interpret the likelihood of abiotic RNA polymerization enhancement in early terrestrial environments that may have harbored the origin of life.

## 2. Materials and Methods

More than two dozen mineral samples including sulfides, oxides, clays, and other silicates were collected for RNA polymerization experiments. With the following noted exceptions, the minerals used in this study were gathered from the in-house collection of the Earth & Environmental Sciences Department of Rensselaer Polytechnic Institute. A montmorillonite-rich clay sample designated Volclay SPV-200 was a gift from the American Colloidal Society. Synthetic faujasite was provided by the Smithsonian Institution (catalog no. NMNH 145899). Quartz was sampled directly for this study from Troy, NY. A polymineralic sample of a black smoker chimney was sampled directly from the Juan de Fuca vent field using the human-occupied vehicle (HOV) *Alvin* during cruise AT15-9 aboard the R/V *Atlantis*. A complete list of the minerals used is presented in Table 1. All other reagents were purchased from Sigma-Aldrich, with the exceptions of sodium chloride and acetonitrile (ACN), which were purchased from VWR International and EMD Millipore, respectively.

All minerals were ground using an agate mortar and pestle. The powders were then passed through a 125 μm sieve and the larger remaining fraction was discarded. The mineralogy of complex natural samples, such as the black smoker chimney, was determined via X-ray powder diffraction (Appendix A). A portion of montmorillonite (Volclay SPV-200) was treated via the Banin process [21,22] and used as a positive control to validate the experimental methods, as RNA oligomers produced in treated montmorillonite experiments are well characterized by a number of independent studies [15,20,56]. Untreated montmorillonite was also included in the experiments reported here in order to assess the ability of naturally occurring clays to enhance polymerization. No other minerals were treated in any way prior to the experiments.

In order to assess the extent of RNA polymerization in these reactions, adenosine 5′-monophosphate was purchased in its free acid form, converted to adenosine 5′-phosphorimidazolide (ImpA) following the protocols described by Pfeffer et al. [57], and used as a representative nucleotide for homopolymeric RNA synthesis.

Experiments were designed and performed to assess RNA polymerization enhancement in aqueous solutions by a variety of different minerals. All experiments were conducted in 0.6 mL nuclease-free microcentrifuge tubes. A stock solution of 15 mM ImpA was prepared using ultrapure water. No other salts were added to this solution.

To avoid potential unwanted chemical interactions and to more closely approximate the chemistry of natural systems, pH buffers were intentionally excluded from these experiments. With few exceptions, the low solubilities of the minerals tested imply a potential for only minor pH deviations. Experiments that include sulfide minerals are more likely than others (e.g., clays, oxides, etc.) to experience a noticeable drop in pH (as is observable in natural systems). An extreme example of such an effect can be observed in natural waters exhibiting acid mine drainage resulting from pyrite oxidation (e.g., [58]). Although it is possible to artificially buffer the pH of an experiment containing any mineral in contact with an aqueous solution, we argue that the more interesting (and realistic) system is one that is allowed to equilibrate without interference from an added buffer. In order to test the quasi-independent effects of pH on oligomerization, we conducted control experiments with ImpA and untreated montmorillonite in the presence of organic pH buffers (pH ~2.5–12) and observed only a slight dampening of oligomer length in acidic solutions (Appendix B). Therefore, although the mineral–nucleotide interactions resulting in enhanced condensation are dependent on pH, they are not a consequence of it. In the non-buffered experiments summarized below, the reported oligomer lengths represent the most conservative (shortest) outcome, and the presence of a pH buffer would have likely resulted in only slightly longer oligomers.

In order to test the ability of halite to enhance polymerization, an additional stock solution of 15 mM ImpA was prepared in a saturated NaCl solution in order to prevent net halite dissolution during the course of the experiment. From these stock solutions, 100 μL was added to each microcentrifuge tube containing 5–6 mg of mineral powder. The microcentrifuge tubes were then sealed and agitated briefly by vortexing before being placed in a dark location and left undisturbed for 3 days. Experiments were conducted at ambient temperature and pressure. All experiments were run alongside a negative control containing 15 mM ImpA and no mineral, as well as a positive control containing 15 mM ImpA and 5–6 mg of treated montmorillonite. An additional negative control containing 15 mM ImpA in a saturated sodium chloride solution with no solid phase was also run alongside experiments containing halite.

Prior to RNA extraction, all run products were centrifuged to remove any suspended mineral particles from the solutions. Oligomer products were then concentrated from the supernatant, de-salted, and extracted using 10 μL C18 ZipTips^®^ from Millipore (Darmstadt, Germany) following the protocols described by Castleberry et al. [59]. A 44 mM ammonium citrate dibasic (AHC) solution was prepared in 50% acetonitrile (ACN) and 50% ultrapure water. This solution was used to dissolve 2,4,6-trihydroxyacetophenone (THAP) in order to make a THAP/AHC matrix solution. The matrix solution was added to each sample in a 1:1 volumetric ratio and vortexed to mix.

Matrix-assisted laser desorption/ionization time-of-flight mass spectrometry (MALDI-TOF MS), a commonly used analytical tool for characterizing abiotic RNA oligomer products [19,20,56,60,61,62], offers superior sensitivity and resolution in addition to precise mass information when compared with other common analytical methods such as HPLC and gel electrophoresis [60,63] and was thus selected for determining the maximum oligonucleotide length formed in each experiment. The advantages of MALDI come at the cost of quantitative data that might allow for calculation of reaction yields. However, given the questions posed in this study, the ability to precisely and accurately determine the extent to which aqueous condensation might occur was prioritized over determining reaction efficiency. To avoid potential pitfalls in MALDI TOF-MS analyses, recommendations by Burcar [63] were taken into account during matrix selection, target selection, and sample preparation. A stainless steel AnchorChip var/387 plate purchased from Bruker Daltonics was used as the MALDI target. The plate held 2 μL of each sample/matrix solution for analysis. In order to promote rapid homogenous crystallization of the matrix, the plate was placed in a 40 °C incubator for approximately 10 min or until no visible fluid phase remained.

**Figure 1:**
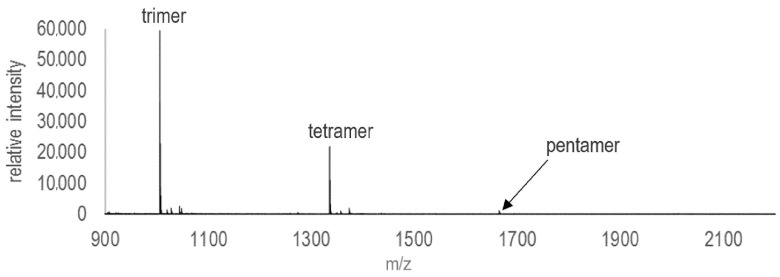
Typical mass spectrum of products analyzed from a 3-day polymerization experiment containing 15 mM ImpA in the presence of 5 mg untreated montmorillonite (SPV-200).

Products were extracted from experiments after three days and analyzed immediately to minimize the degradation of oligomers. All experiments were analyzed on an Autoflex Speed MALDI-TOF MS manufactured by Bruker (Billerica, MA, USA) made available by the Rensselaer CBIS Proteomics Core Facility (Troy, NY, USA). Instrument settings are shown in Table 2. Prior to the collection of spectra, the instrument was externally calibrated with Oligonucleotide Calibration Standard LMW (reference 8217028) purchased from Bruker Daltonics with a covered mass range of ~1000 Da to 4000 Da. In addition to performing baseline subtractions, Savitsky–Golay polynomial regression was used to smooth all collected spectra. A typical mass spectrum collected via this method is shown in Figure 1. For each set of experimental conditions, the maximum detected oligomer lengths were determined from the spectra. For each set of conditions, triplicate experiments were analyzed and the maximum lengths detected in each spectrum were averaged.

## 3. Results

**Figure 2:**
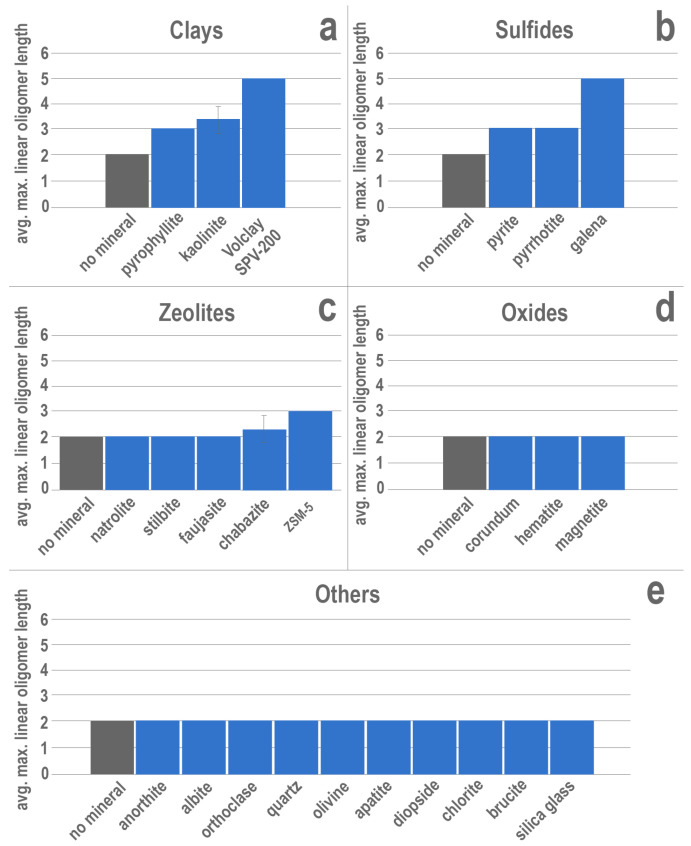
Results of mineral experiments containing untreated (**a**) clay minerals, (**b**) sulfide minerals, (**c**) zeolites, (**d**) oxide minerals, or (**e**) other solid phases. Vertical-axis values represent the average maximum linear oligomer length detected via MALDI-TOF MS among triplicate experiments. Error bars represent one standard deviation from the average maximum length. Absence of error bars indicates no variation between triplicate experiments. Negative control experiments containing no mineral are shown in grey.

Enhanced ribonucleotide polymerization was detected in the presence of several different mineral phases among the clays, sulfides, and zeolites (Figure 2). All three clay mineral experiments exhibited enhanced polymerization, producing longer oligomers than those detected in negative controls with no mineral phase (Figure 2a). All results are reported as maximum oligomer length (in number of nucleotide subunits) detected in mass spectra, averaged across triplicate experiments. A typical spectrum is shown in Figure 1. Negative controls (15 mM ImpA, no mineral) consistently produced nothing longer than dimers. Experiments that contained untreated montmorillonite consistently produced pentamers. RNA polymerization was also promoted in the presence of kaolinite, with an average maximum linear oligomer length of 3.33 nucleotides, as well as pyrophyllite, which produced trimers.

In addition to the clay minerals, all the sulfide minerals tested also produced oligomers longer than the dimers detected in negative controls (no mineral phase; Figure 2b). Of these minerals, experiments containing galena yielded the longest linear oligomers (pentamers), matching those observed in untreated montmorillonite experiments. Additionally, the iron sulfides, pyrite and pyrrhotite, each consistently produced trimers from the activated nucleotides. Two of the zeolites tested also showed enhanced polymerization. The synthetic zeolite, ZSM-5, consistently promoted the formation of trimers, whereas chabazite showed a minor increase in the average maximum linear oligomer length (2.33 nucleotides) relative to the negative controls (2.00 nucleotides). Experiments containing natrolite, stilbite, and synthetic faujasite all yielded dimers and did not further enhance polymerization. The remainder of the minerals tested showed no enhanced polymerization beyond dimerization (Figure 2d–e).

**Figure 3:**
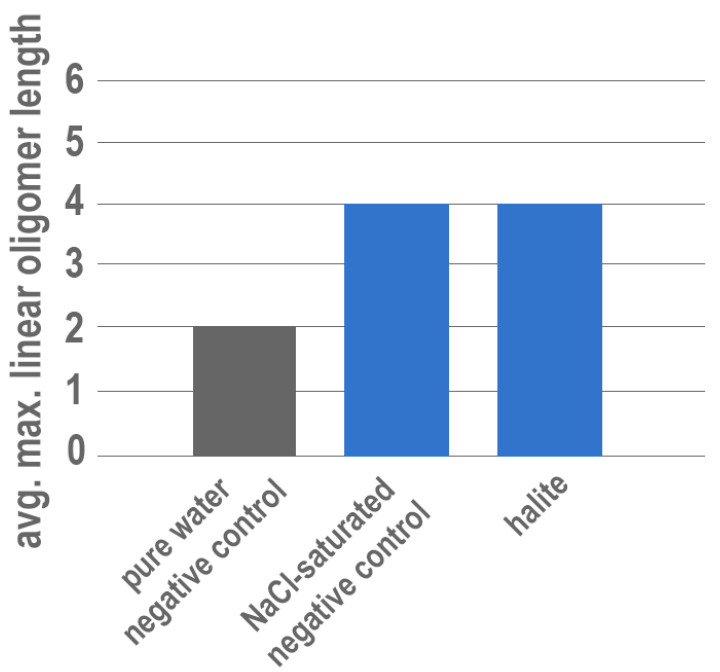
Results of experiments containing solutions saturated with NaCl in the presence and absence of halite compared with negative control experiments conducted in pure water. Negative control conducted with ultrapure water is shown in grey. Experiments conducted in a NaCl-saturated solution are shown in blue.

Finally, an attempt was made to investigate the potential for salts (e.g., metal chlorides) to also enhance polymerization; halite (NaCl) was chosen to represent the chloride evaporites. However, because halite is highly soluble, it was not possible to conduct an experiment with 15 mM ImpA in ultrapure water because the powdered mineral readily dissolves, complicating the potential for surface catalysis. Instead, halite experiments were conducted in a NaCl-saturated solution with 15 mM ImpA to prevent net dissolution. Control experiments with no solid mineral phase were also conducted in the NaCl-saturated solution for comparison. However, enhanced polymerization resulting in consistent tetramer formation was observed in all NaCl-saturated solutions, including those with no solid halite present (Figure 3).

## 4. Discussion

Experiments conducted in the presence of brucite, olivine, hematite, or magnetite showed no difference in products compared with the negative control experiments lacking a solid mineral phase; these results are consistent with earlier findings from Miyakawa et al. [55]. Similarly, no effects on polymerization were observed when natrolite, stilbite, faujasite, corundum, anorthite, albite, orthoclase, quartz, olivine, apatite, diopside, chlorite, brucite, or amorphous silica glass were included in the experiments. The long list of minerals that exhibit no observable effect on polymerization demonstrates that interactions between potential organic reactants and inorganic mineral substrates are far from ubiquitous processes. Minerals that do positively influence condensation are therefore potentially valuable tools for both fostering prebiotic chemistry and for assessing the feasibility of such chemistry in associated environments.

Experiments yielding tetramers of RNA from solutions saturated in NaCl with and without solid halite are somewhat surprising but have a direct connection to a specific type of environment—namely those in which highly saline waters can form due to isolation of seawater and subsequent evaporation resulting in the concentration of aqueous salts (and presumably of organic reactants). These results also show that the previously documented effects of aqueous metals on polymerization (in the absence of a solid phase) [14,64,65] also extend to Na^+^ at high enough concentrations. As ancient oceans were likely rich in sodium, the influence of aqueous Na^+^ over polymerization and the apparent dependency on concentration may have important implications for prebiotic chemistry and will be discussed in detail in a future publication.

In addition to NaCl-saturated experiments, the positive results from experiments conducted in this study that contained minerals demonstrate that various clay minerals, sulfide minerals, and zeolites are also capable of enhancing polymerization of RNA abiotically in aqueous solutions. There is significance in the simple fact that multiple minerals other than montmorillonite have now been demonstrated to promote RNA polymerization in aqueous systems. Supplementing observations made by Burcar et al. [51] at high pressures, experiments conducted in this study reveal that a number of other minerals and mineral classes are also capable of enhancing abiotic polymerization of RNA at 1 bar. These results suggest that oligomeric RNA can feasibly accumulate in a variety of natural systems without relying on a specific artificially optimized montmorillonite for the reaction.

Experiments conducted with untreated montmorillonite show that chemical treatment of the mineral is not necessary to promote polymerization; the potential role of montmorillonite in a prebiotic setting on the early Earth consequently becomes more feasible. The convention of treating montmorillonite prior to conducting synthesis experiments might suggest that the conditions at which condensation is facilitated by the solid mineral phase are specific and exclusive. However, the results presented here show that the positive influence of montmorillonite (and more broadly non-clay minerals) over elongation of oligoribonucleotides is much more general and does not require extraordinary circumstances. This may come at the expense of efficiency, but there is no obvious reason to believe that the processes leading to the origin of life on the early Earth were efficient or optimized in any way. The synthesis of life was not a goal of geological processes but rather a consequence of a planet born far from equilibrium. It is thus prudent to consider the state of the early Earth *first*, then consider the processes allowed for within that state when searching for the pathways to life.

Elongated oligomers detected in the presence of the other tested clay minerals, kaolinite and pyrophyllite, further relax the constraints of clay properties necessary for enhancement of abiotic polymerization. For example, pyrophyllite is similar in structure to montmorillonite, possessing two tetrahedral silicate layers that surround each octahedral hydroxide layer (2:1), but lacks a layer charge [33]. Therefore, the observation of polymerization enhancement in the presence of pyrophyllite suggests that the negative structural charge exhibited by montmorillonite is not exclusively necessary for promoting polymerization. Kaolinite deviates even further from the properties and structure of montmorillonite, containing a single tetrahedral silicate layer for each octahedral hydroxide layer [66]. The enhanced oligomerization observed in the presence of kaolinite in this study demonstrates that, like surface charge, the dioctahedral 2:1 nature of both montmorillonite and pyrophyllite is not a requirement for promotion of oligonucleotide condensation via a clay mineral. Furthermore, neither pyrophyllite nor kaolinite exhibit the swelling behavior characteristic of smectites [67,68].

Both pyrophyllite and kaolinite are commonly produced via the weathering of minerals more felsic than those that produce montmorillonite. These clay minerals could have been available, given the presence of felsic crust in the Hadean [31,32], and may have increased in abundance as the Earth evolved [33]. These results should also encourage investigation of other clay and non-clay minerals, as they demonstrate that montmorillonite is not uniquely suited to fostering prebiotic condensation reactions, thus broadening the possible environments in which these reactions could have occurred on the early Earth.

Alongside the clay minerals tested in this study, several experiments containing zeolites also produced RNA oligomers that exceeded the dimers observed in negative controls. A variety of zeolites, including faujasite and the synthetic Al-poor silicalite, have previously been suggested as likely candidates for the catalysis of condensation reactions in aqueous solutions based on their large capacities for adsorption and their hydrophobic/organophilic tendencies [50]. Whereas the synthetic faujasite tested in this study did not seem to have any effect on the lengths of oligomers produced, experiments containing ZSM-5 consistently produced trimers of RNA, thus demonstrating that zeolites are indeed capable of promoting the condensation reaction, though apparently to a modest extent at the tested conditions. In addition to ZSM-5, trimers were detected in two out of three replicate experiments containing chabazite. However, it is not clear if this is the result of weak interactions between the mineral and the nucleotides or if it is due to variation in the purity of activated nucleotide between replicates. Although polymerization was not observed to the same extent in experiments containing natural zeolite minerals as it was in those conducted with ZSM-5, many organophilic zeolites remain untested and may be capable of reproducing the behavior observed in experiments containing synthetic ZSM-5. Zeolites are common products of low-grade metamorphism of volcanic glasses of varying compositions [69] and thus could have been abundantly available early in Earth’s history. Their exceptional capacity for adsorption, combined with their probable abundance on the early Earth makes them promising candidates for hosting a variety of prebiotically relevant reactions, not just oligoribonucleotide synthesis. Despite this, zeolites have garnered comparatively little attention in prebiotic chemistry experiments and thus warrant more extensive investigation beyond the scope of this study. Nevertheless, the results of this study successfully demonstrate experimentally that zeolites have the capacity to positively influence abiotic synthesis of biologically relevant molecules as previously proposed [49,50,70].

The structures of both zeolites and clay minerals allow for the possibility of organic constituents to be sequestered and concentrated internally, whether within the layers of bentonites or organophilic zeolitic pores. In contrast, however, sulfide minerals lack a means of accommodating monomers within their internal structure. Nevertheless, all the sulfide minerals explored in this study positively impacted the maximum RNA oligomer length detected in their respective experiments. Sulfide minerals, including pyrite, pyrrhotite, and galena are common products of hydrothermal interactions with host rocks of varying compositions and would have been abundant following the onset of plate tectonics [23]. Preceding plate tectonics, it is possible that these minerals may have been produced in other hydrothermal environments, including impact-generated hydrothermal systems and hot spot volcanism. Prebiotic precipitation of pyrite via the reaction of aqueous ferrous iron with H_2_S has been proposed as the first energy source for life due to its highly exergonic nature [71]. Wächtershäuser posited that the polyanionic structure of RNA would result in the polymer being preferentially bound to the surface of pyrite [72], and adsorption studies confirm that pyrite efficiently adsorbs RNA [53], as well as adenosine 5′-monophosphate and other phosphorylated molecules [73]. Similarly, pyrrhotite has more recently been shown to adsorb RNA from solution [74]. The results presented here show that pyrite and pyrrhotite can not only concentrate RNA but also facilitate its condensation.

Alongside the iron sulfides, galena was also tested and found to be the most effective of the sulfide minerals at enhancing polymerization, matching montmorillonite in apparent effectiveness and consistently yielding pentamers of RNA. Miyakawa et al. previously reported galena as an effective catalyst for RNA polymerization, though only dimerization of adenosine 5′-phosphorimidazolide was observed in analogous experiments [55]. That study further emphasized that galena is inferior to montmorillonite in terms of its catalytic ability. However, this comparison was made using Na^+^-montmorillonite after optimizing Volclay SPV-200 via the Banin process. In contrast, the results presented here show that galena exhibits catalytic behavior to the same (or similar) extent as naturally occurring montmorillonite clay (as would be present on the early Earth). It has been reported that adding dissolved lead (Pb^2+^) is an effective means of synthesizing RNA oligomers from activated monomers [65,75,76]. It is therefore possible that introduction of the aqueous lead via dissolution of galena may be responsible for the longer oligomers detected in galena-bearing experiments. However, Miyakawa et al. demonstrated that there was no catalytic effect from the supernatant extracted from the presence of galena; dimerization was only observed when the solid phase was also present [55]. The mechanism by which galena promotes the elongation of oligoribonucleotides is therefore more likely attributable to mineral surface interactions and analogous to the similar interactions between RNA and iron sulfide minerals discussed above.

These experiments demonstrate the degree of complexity surrounding polymerization reactions due to the multivariable effects of the environment in which they may occur. Both mineral and fluid chemistry must be considered in order to effectively assess the potential for RNA polymerization in prebiotic environments, and multiple reaction mechanisms must be examined. By isolating a single variable and testing an array of minerals, this study demonstrates that RNA polymerization (and polymerization reactions generally) may be enhanced in a wide variety of environments and is not limited to just those containing montmorillonite clay. Admittedly, all the experiments reported in this study (and a large majority of other RNA polymerization investigations) rely heavily on the activating power of imidazole to fuel the reaction; it is only after the ribonucleotides are pre-activated that any elongation is detectable. However, imidazole is only implemented in this study as an analog for a prebiotic energy source in order to assess how condensation could be influenced by various mineral phases in various environments and for ease of comparison between the results presented here and those of previous studies. It was not selected on a basis of prebiotic relevance, nor was it a goal of this study to identify alternative sources of chemical energy that may have been available to aid the development of life on the early Earth. Further investigation into the matter is certainly warranted; the reactions studied here may behave differently if the observed interactions rely heavily on the nature of the energy source utilized (i.e., imidazole). However, investigating and characterizing alternative prebiotic energy sources is beyond the scope of this study. Rather, it is the aim of this study to broaden the scope of investigation of the interactions between prebiotically relevant organic molecules and the inorganic mineral phases that could have hosted emerging life. Montmorillonite is not an exclusive solution to the challenge of polymerization in aqueous environments, nor are montmorillonite-associated environments the only potential sites that allow for productive prebiotic chemistry. It is shown here that a variety of other minerals and mineral types, including other clays, zeolites, and sulfides also influence oligomer synthesis, thus showing that a wider range of environments and pathways should be considered going forward.

## Figures and Tables

**Table 1:** Minerals tested in this study for their ability to enhance oligoribonucleotide condensation.

Mineral Name	Mineral Type	General Formula
Montmorillonite (Volclay SPV-200)	Clay (Phyllosilicate)	(Na, Ca)_0.33_(Al, Mg)_2_(Si_4_O_10_)(OH)_2_·nH_2_O
Pyrophyllite	Clay (Phyllosilicate)	Al_2_Si_4_O_10_(OH)_2_
Kaolinite	Clay (Phyllosilicate)	Al_2_Si_2_O_5_(OH)_4_
Nontronite	Clay (Phyllosilicate)	(CaO_0.5_, Na)_0.3_Fe^3+^_2_(Si, Al)_4_O_10_(OH)_2_·nH_2_O
Pyrite	Sulfide	FeS_2_
Pyrrhotite	Sulfide	Fe_1−x_S (x = 0 to 0.2)
Galena	Sulfide	PbS
Natrolite	Zeolite	Na_2_Al_2_Si_3_O_10_·2H_2_O
Stilbite	Zeolite	NaCa_4_(Si_27_Al_9_)O_72_·28(H_2_O)
Faujasite (synthetic)	Zeolite	(Na_2_, Ca, Mg)_3.5_[Al_7_Si_17_O_48_]·32(H_2_O)
Chabazite	Zeolite	(Ca, K_2_, Na_2_)_2_[Al_2_Si_4_O_12_]_2_·12H_2_O
ZSM-5 (synthetic)	Zeolite	Na_n_Al_n_Si_96–n_O_192_·16H_2_O (0 < n < 27)
Corundum	Oxide	Al_2_O_3_
Hematite	Oxide	Fe_2_O_3_
Magnetite	Oxide	Fe^2+^Fe^3+^_2_O_4_
Anorthite	Feldspar (Tectosilicate)	CaAl_2_Si_2_O_8_
Albite	Feldspar (Tectosilicate)	NaAlSi_3_O_8_
Orthoclase	Feldspar (Tectosilicate)	KAlSi_3_O_8_
Quartz	Tectosilicate	SiO_2_
Olivine	Nesosilicate	(Mg, Fe)_2_SiO_4_
Black Smoker Chimney	(Polymineralic)	N/A
Apatite	Phosphate	Ca_5_(PO_4_)_3_(F, Cl, OH)
Diopside	Pyroxene (Inosilicate)	MgCaSi_2_O_6_
Chlorite	Phyllosilicate	(Fe, Mg, Al)_6_(Si, Al)_4_O_10_(OH)_8_
Brucite	Hydroxide	Mg(OH)_2_
Silica (synthetic)	(Glass)	SiO_2_

**Table 2:** MALDI-TOF MS instrument settings used for Bruker Autoflex Speed.

Instrument Parameter	Value
Detection Mode	Reflectron
Ionization Mode	Negative
Ion Source 1	19 kV
Ion Source 2	16.7 kV
Lens	8.4 kV
Pulsed Ion Extraction	140 ns
Relative Laser Intensity	75–85%
Sets of Shots Per Sample	5
Shots Per Set	1000

**Figure A1:**
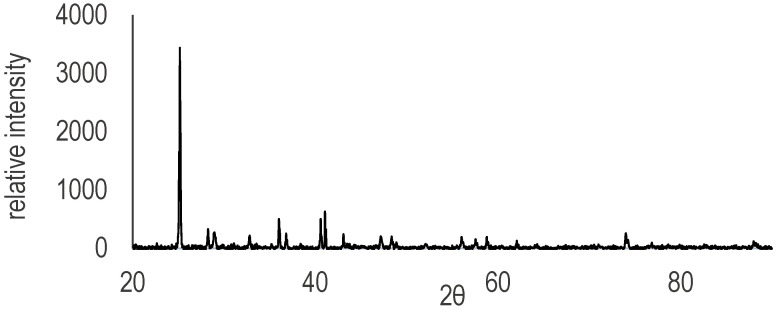
X-ray diffraction spectrum of powdered polymineralic black smoker sample indicating the likely presence of sulfate and sulfide minerals.

**Figure A2:**
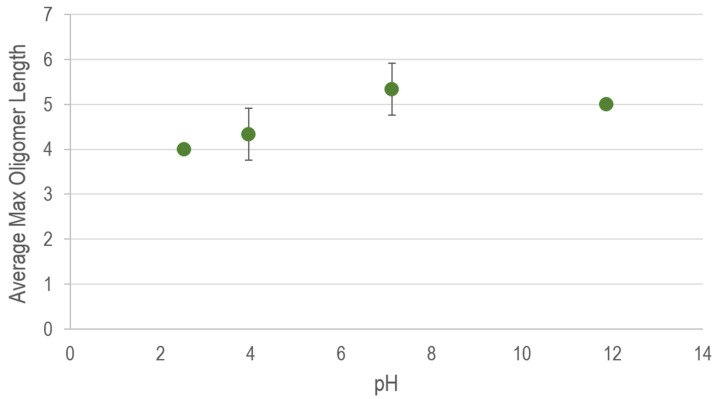
Maximum linear oligomer lengths detected in pH-buffered control experiments containing 15 mM ImpA and approximately 5 mg Volclay SPV-200. Vertical-axis values represent the average maximum linear oligomer length detected via MALDI-TOF MS among triplicate experiments. Error bars represent one standard deviation from the average maximum length. Absence of error bars indicates no variation between triplicate experiments.

## Data Availability

Data available upon request.
